# MicroRNAs Participate in Morphological Acclimation of Sugar Beet Roots to Nitrogen Deficiency

**DOI:** 10.3390/ijms25169027

**Published:** 2024-08-20

**Authors:** Xinyu Liu, Zhenqiang Lu, Qi Yao, Lingqing Xu, Jingjing Fu, Xilong Yin, Qing Bai, Dali Liu, Wang Xing

**Affiliations:** 1 Province Key Laboratory of Plant Gene and Biological Fermentation in Cold Regions, College of Life Science, Heilongjiang University, Harbin 150080, China; xinyu_liu@163.com (X.L.); zhenqianglu@163.com (Z.L.); 15714511208@163.com (Q.Y.); 2 National Beet Medium-Term Gene Bank, Heilongjiang University; Harbin 150080, China; lingqingxu98@163.com (L.X.); bling199904@163.com (J.F.); xilongyin_hlju@163.com (X.Y.); bq2375971288@163.com (Q.B.); 3 Key Laboratory of Sugar Beet Genetics and Breeding, College of Advanced Agriculture and Ecological Environment, Heilongjiang University, Harbin 150080, China

**Keywords:** nitrogen deficiency, *Beta vulgaris*, root, MicroRNAs, miR156a

## Abstract

Nitrogen (N) is essential for sugar beet (*Beta vulgaris* L.), a highly N-demanding sugar crop. This study investigated the morphological, subcellular, and microRNA-regulated responses of sugar beet roots to low N (LN) stress (0.5 mmol/L N) to better understand the N perception, uptake, and utilization in this species. The results showed that LN led to decreased dry weight of roots, N accumulation, and N dry matter production efficiency, along with damage to cell walls and membranes and a reduction in organelle numbers (particularly mitochondria). Meanwhile, there was an increase in root length (7.2%) and branch numbers (29.2%) and a decrease in root surface area (6.14%) and root volume (6.23%) in sugar beet after 7 d of LN exposure compared to the control (5 mmol/L N). Transcriptomics analysis was confirmed by qRT-PCR for 6 randomly selected microRNAs, and we identified 22 differentially expressed microRNAs (DEMs) in beet root under LN treatment. They were primarily enriched in functions related to binding (1125), ion binding (641), intracellular (437) and intracellular parts (428), and organelles (350) and associated with starch and sucrose metabolism, tyrosine metabolism, pyrimidine metabolism, amino sugar and nucleotide sugar metabolism, and isoquinoline alkaloid biosynthesis, as indicated by the GO and KEGG analyses. Among them, the upregulated miR156a, with conserved sequences, was identified as a key DEM that potentially targets and regulates squamosa promoter-binding-like proteins (SPLs, 104889216 and 104897537) through the microRNA-mRNA network. Overexpression of miR156a (MIR) promoted root growth in transgenic Arabidopsis, increasing the length, surface area, and volume. In contrast, silencing miR156a (STTM) had the opposite effect. Notably, the fresh root weight decreased by 45.6% in STTM lines, while it increased by 27.4% in MIR lines, compared to the wild type (WT). It can be inferred that microRNAs, especially miR156, play crucial roles in sugar beet root’s development and acclimation to LN conditions. They likely facilitate active responses to N deficiency through network regulation, enabling beet roots to take up nutrients from the environment and sustain their vital life processes.

## 1. Introduction

Nitrogen (N) is one of the essential mineral nutrients required for plants, and it participates extensively in various activities within organisms [1]. Over the past few decades, in order to achieve increased crop production, the use of nitrogen fertilizer has continuously increased, and it is expected to reach 2.4 million tons by 2050 [2]. Unfortunately, approximately 50–70% of N is not used by plants [3]. The root’s structure and its absorption activity influence N uptake and assimilation in plants, and root growth and development in response to local and systemic N signals play crucial roles in N regulation [4]. In addition, as an important organ in plants, the root system can also adapt to adverse conditions by modifying its morphology [5]. Sufficient N can certainly maximize the N uptake by the root system, but under conditions of limited N supply, plants can also maintain necessary growth and development by elongating their lateral roots to improve their N acquisition efficiency [6]. In addition, the growth of the aboveground parts of the plants also largely depends on the ability of the roots to absorb nutrients from the soil. Under low N (LN) conditions, aboveground growth is inhibited, and more carbohydrates are allocated to the root system, which in turn promotes root growth and maintains the balance of carbon and nitrogen metabolism in plants [7,8]. Thus, it has been reported that under mild nitrogen deficiency, maize adopted a positive strategy by increasing its root biomass, total root length, root surface area, and number of lateral roots, while decreasing the average root diameter [9,10]. Moreover, it is found that N deficiency leads to ultrastructural changes such as severe damage of the chloroplast structure [11].

Efficient N use enables plants to effectively address low-N supply issues, but this regulatory process is highly complex, involving microRNAs as one of the key regulatory factors [12]. MicroRNAs, a class of endogenous non-coding RNAs of typically 18 and 24 nt, were first identified in *C. elegans* [13] and have since been found in many plants. In recent years, advancements in high-throughput sequencing technology have made transcriptomics a common approach for identifying microRNAs in plants under environmental stress, including LN [14,15]. It is reported that miR167a expression was downregulated under LN conditions, resulting in the upregulation of its target gene, *auxin response factor 8* (*ARF8*), which may promote the relative stability of the N level in plants [16,17]. In *Arabidopsis thaliana*, under LN stress, miR156 expression was upregulated, while miR169, miR397, miR398, and miR827 were downregulated [18]. In rice, Shin et al. found that three-quarters of the microRNAs identified from root transcriptomes under N starvation were downregulated, with osa-miR169 family members being the most significant [19].

Sugar beet (*Beta vulgaris* L.) is the second largest sugar crop globally and in China, and it is a biennial plant with self-incompatibility. In addition to sugar production, it has diverse applications including betaine extraction and animal feed [20]. As a crop with high N demands, sugar beet’s growth and development are closely associated with the N supply [21]. Although extensive N application enhances the taproot and sugar yield, thus increasing economic returns, excessive N can also lead to soil compaction, water eutrophication, and other severe environmental issues, negatively impacting the sustainable development of sugar beet [22]. Hence, elucidating the physiological mechanisms of sugar beet under LN supply and revealing important LN response patterns are crucial for improving N use efficiency. The root system, as the primary organ interacting with N in the soil, significantly influences plants’ tolerance to nutrition stress [23]. Thus, it is necessary to analyze the functional strategies of sugar beet roots in response to an LN environment. In this study, we investigated the effects of LN stress on beet roots’ morphology, N accumulation, and ultrastructure. Through transcriptomic analysis, we identified microRNAs associated with LN stress to elucidate how these molecules mediate adaptive changes in beet roots.

## 2. Results

### 2.1. Effects of Low-Nitrogen Stress on Root Morphology of Sugar Beet Seedlings

**Figure 1:**
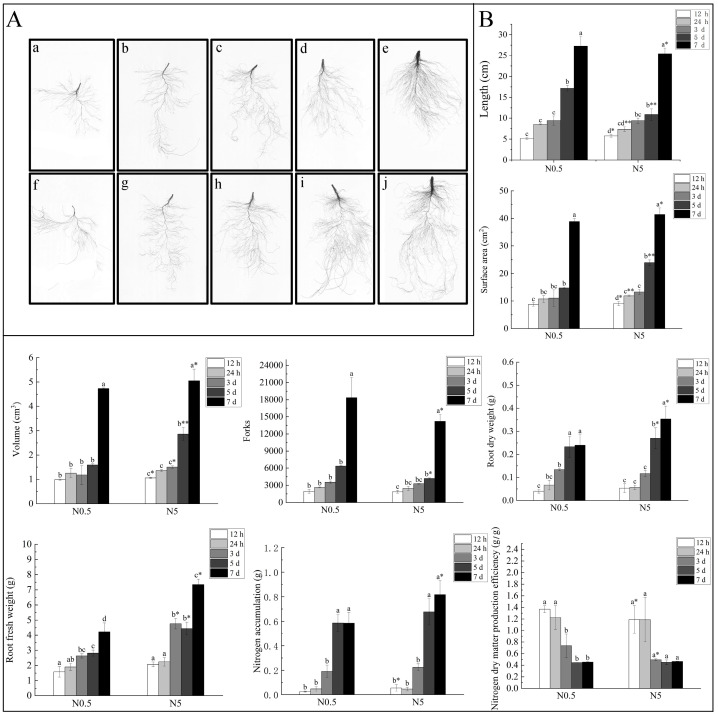
Phenotypic performance and N content of sugar beet roots subjected to LN stress. (**A**) a–e and f–j indicate morphology and structure of roots under N0.5 or N5 (control) at 12 h, 24 h, 3 d, 5 d, and 7 d, respectively. (**B**) Determination of beet root growth and N content. Lowercase letters indicate significance at same nitrogen level (*p* < 0.05). Asterisks denote significant differences between nitrogen levels at same time point, with * for *p* < 0.05 and ** for *p* < 0.01.

Roots serve as the primary organs for plants to absorb nutrients. Assessing the morphological performance of beet roots can visually demonstrate their response to N deficiency. As shown in Figure 1A, LN stress (0.5 mmol/L N) notably affected sugar beet roots. Data from root scanning revealed increasing differences between the LN and control conditions (normal N, 5 mmol/L N) with prolonged stress duration. By the 7th day, compared to the control, the root length and branch number under LN increased by 7.2% and 29.2% (*p* < 0.05), respectively; the root surface area and root volume decreased by 6.14% and 6.23% (*p* < 0.05), respectively, indicating that LN induced root elongation and lateral root growth. Under LN conditions, the root fresh/dry weight and nitrogen accumulation in sugar beets were consistently lower than in the controls, and the N dry matter production efficiency decreased with extended stress duration, demonstrating the impact of LN on biomass accumulation in sugar beet roots (Figure 1B). In conclusion, LN promoted root growth, enhanced root density, and improved N uptake efficiency. It is noteworthy that during the initial stages of LN stress, these indicators showed minimal changes, which rapidly escalated starting from the 5th day, indicating that phenotypic differences occurred only when a certain threshold of N deficiency in sugar beet was reached.

### 2.2. Ultrastructural Changes in Sugar Beet Roots under Low-Nitrogen Stress

**Figure 2:**
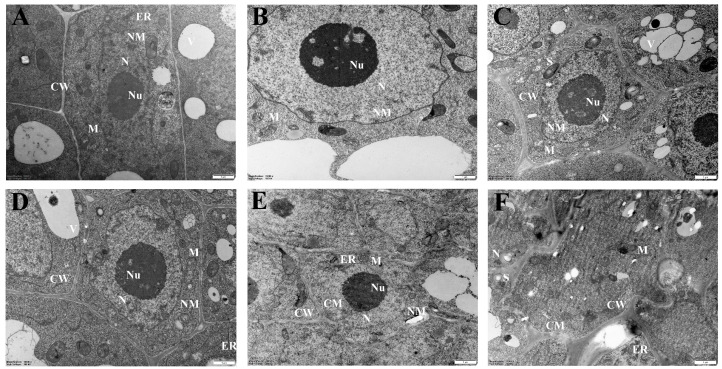
Sugar beet roots’ ultrastructure under LN stress. (**A**–**F**) represent 0 h (control), 12 h, 24 h, 3 d, 5 d, and 7 d of LN treatments, respectively. CW—cell wall; CM—cell membrane; N—nucleus; Nu—Nucleolus; NM—Nuclear Membrane; M—mitochondrion; S—starch grain; V—Vacuole; ER—Endoplasmic Reticulum; The scale bar is 1 μm, ×15,000.

To gain further insights into the effects of LN stress on sugar beet roots’ cell physiology, we conducted observations on the ultrastructure of the roots. As shown in Figure 2, the control group exhibited a normal morphology of root tip cells, with intact cell walls, cell membranes, and clear nuclei and rounded or oval mitochondrial structures. Conversely, prolonged exposure to LN resulted in gradual cell wall shrinkage, enlarged intercellular spaces, the disappearance of membrane structures, and fragmentation and reduced numbers of organelles such as mitochondria. By the 7th day of treatment, membrane structures were no longer discernible, indicating significant cellular damage.

### 2.3. MicroRNA Identification after RNA-Seq in Sugar Beet Roots under Low-Nitrogen Stress

**Figure 3:**
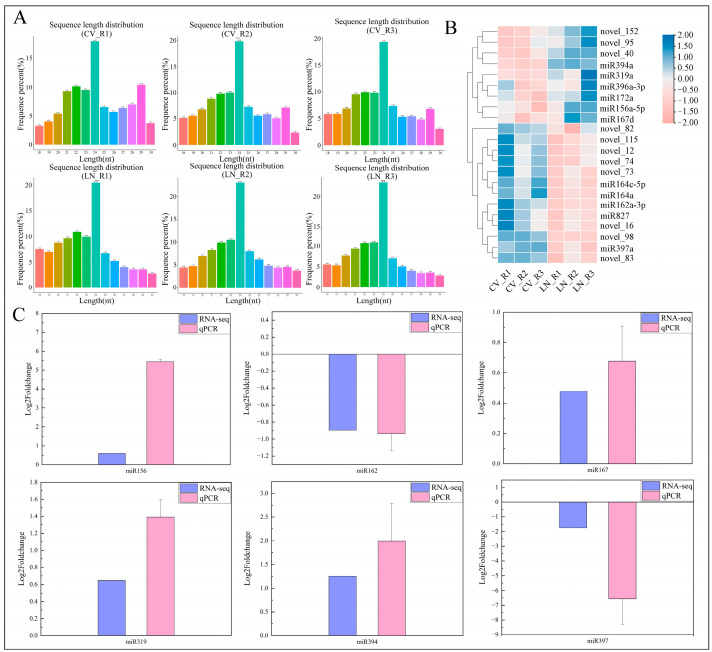
MicroRNA sequence length distribution (**A**), clustering heat map of differentially expressed microRNAs (**B**), and transcriptome data validation by qRT-PCR (**C**) in beet roots under LN. CV_R represents control group (5 mol/L N), while LN_R indicates LN group (0.5 mol/L N). R1–R3 denote three biological replicates. The clustering method used is log_10_ for TPM value of each group, with blue indicating upregulation and pink indicating downregulation.

Beet roots subjected to LN and normal N (CV) conditions were collected in triplicate for RNA extraction, library construction, and RNA-Seq analysis. From the six cDNA libraries, a total of 16,258,407, 14,578,867, and 11,021,095 reads were obtained from the three libraries of CV_R, while 13,207,730, 12,272,843, and 15,041,915 reads were obtained from LN_R. The quality assessment of the sequenced fragments revealed error rates below 0.01%, with Q20 > 99.5%, Q30 > 98.5%, and GC content > 49.5%. Subsequently, clean data (Table S1) were obtained by removing adapter sequences and low-quality reads, and microRNAs were selected from the clean reads. As shown in Figure 3A, the length distribution of microRNAs in beet root was predominantly 24 nt, with a range (18–30 nt) that was similar to those in other plants, indicating the reliability of the sequencing data.

A total of 126 microRNAs were identified in the six libraries. Among them, 22 microRNAs exhibited differential expressions in sugar beet roots under LN stress, including 11 known microRNAs and 11 novel microRNAs. Specifically, 9 of them were upregulated (miR167d, miR396a-3p, miR156a-5p, miR319a, miR172a, novel_95, miR394a, novel_152, novel_40), while 13 showed downregulation (miR397a, novel_98, novel_83, miR827, novel_82, novel_73, novel_74, miR162a-3p, miR164c-5p, novel_16, miR164a, novel_115, novel_12) (Figure 3B).

We randomly selected six different microRNAs to validate the transcriptome data. As shown in Figure 3C, the microRNA transcriptome sequencing data (RNA-seq) and qRT-PCR results exhibited a consistent trend. However, there were differences in the degree of upregulation or downregulation, which might be attributed to potential differences in sensitivity between the two methods.

### 2.4. GO and KEGG Enrichment Analysis of Differentially Expressed microRNAs under Low-Nitrogen Stress

**Figure 4:**
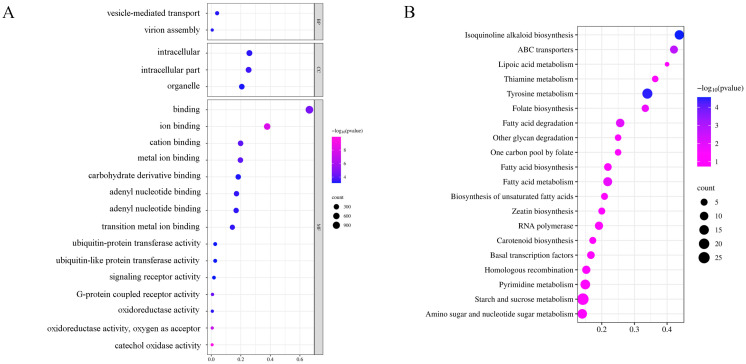
GO (**A**) and KEGG (**B**) enrichment of DEMs in sugar beet roots under LN treatment. The X-axis represents the enrichment factors and q value-log_10_, the left side of the Y-axis shows the GO or KEGG pathway names, and the right side indicates the GO classification. The dot size represents the number of enriched microRNAs.

We conducted enrichment analysis of differentially expressed microRNAs (DEMs) in sugar beet roots under LN stress to elucidate their biological functions. DEMs with a significance level of *p* < 0.05 were selected for pathway analysis. As shown in Figure 4A, most of the DEMs were associated with molecular functions, while a smaller proportion was linked to biological processes and cellular components. DEMs in the top five enriched functions were found to participate in binding (1125), ion binding (641), intracellular (437) and intracellular parts (428), and organelles (350). These findings suggest that DEMs play important roles in root cells, particularly in intracellular transport processes and stress response mechanisms. Additionally, KEGG enrichment analysis identified significant pathways such as starch and sucrose metabolism, tyrosine metabolism, pyrimidine metabolism, amino sugar and nucleotide sugar metabolism, and isoquinoline alkaloid biosynthesis (Figure 4B). These results indicate that sugar beet roots may respond to a LN supply by altering the expression of microRNAs that are involved in sugar synthesis and metabolism, as well as secondary metabolism.

### 2.5. Regulation of Differentially Expressed microRNAs Correlated with Target Genes

**Figure 5:**
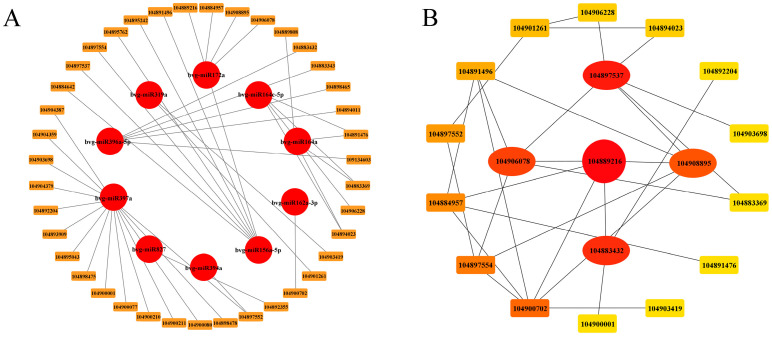
The microRNA regulatory network ((**A**), red circles indicate DEM names, orange squares indicate DEM-regulated target genes) and protein–protein interaction (PPI) network of the DEM target genes ((**B**), genes are represented by nodes, with lines indicating interactions between genes; the redder the color is, the stronger the interaction is).

To further elucidate the potential roles of DEMs in relation to their target genes, Targetfinder was introduced to predict targets for 10 known microRNAs, and a total of 37 genes were screened (Figure 5A). Subsequently, these genes were analyzed using the STRING database, and non-connected nodes were filtered out to construct a regulatory network visualized in Cytoscape (Figure 5B). The results highlighted five core genes: 104889216, 104897537, 104883432, 104906078, and 104908895. Specifically, 104889216 and 104897537 were identified as target genes of miR156a-5p, encoding squamosa promoter-binding-like protein (SPL); 104883432 was found to be targeted by miR396a-5p, which encodes growth-regulating factor (GRF); and 104906078 and 104908895 were targets of miR172a, encoding AP2-like ethylene-responsive transcription factor TOE3 (AP2). According to the GO and KEGG analysis, miR156a-5p and its target genes may play a significant role in sugar beet roots’ response to LN stress.

### 2.6. Sequence Analysis of miR156a Mature Bodies

**Figure 6:**
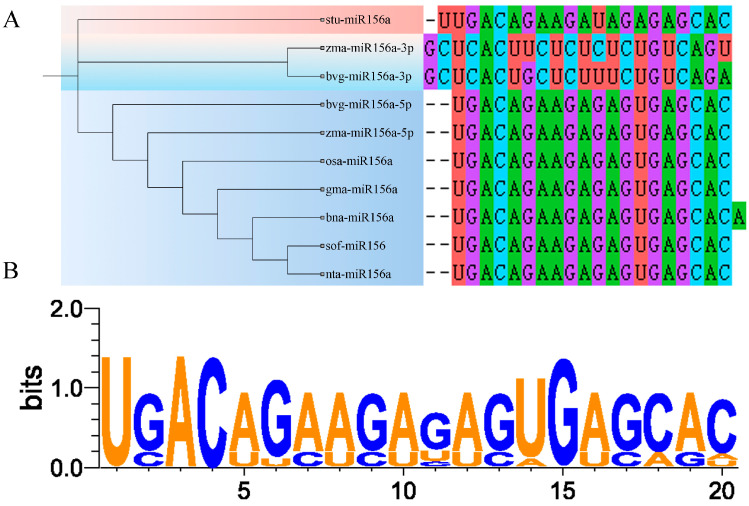
Sequence analysis of miR156a mature bodies. (**A**) Phylogenetic tree of miR156a mature sequences across each species. (**B**) Conserved sequence diagram.

Next, an evolutionary tree of miR156a mature sequences from beet and seven other species was constructed, and it can be seen from Figure 6A that bvg-miR156-3p exhibited a close phylogenetic relationship with zma-miR156-3p. Additionally, bvg-miR156-5p shared a highly conserved sequence pattern with sequences from other species (Figure 6B), indicating its relative conservation across different species.

### 2.7. Generation and Identification of Transgenic Arabidopsis thaliana

**Figure 7:**
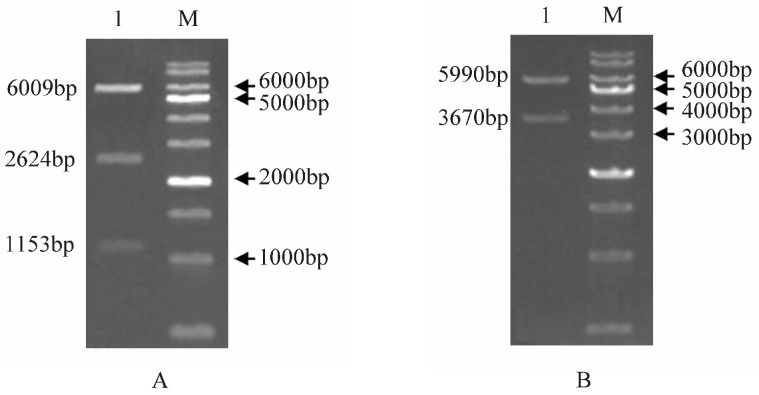
Vector construction of pCAMBIA1300-35S-bvg-MIR156a with the *EcoR* V cleavage site (**A**) and pCAMBIA1300-35S-bvg-STTM-miR156a with the *Age* I cleavage site (**B**). M, 1000 bp DNA marker; lane 1, digested DNA fragments.

**Figure 8:**
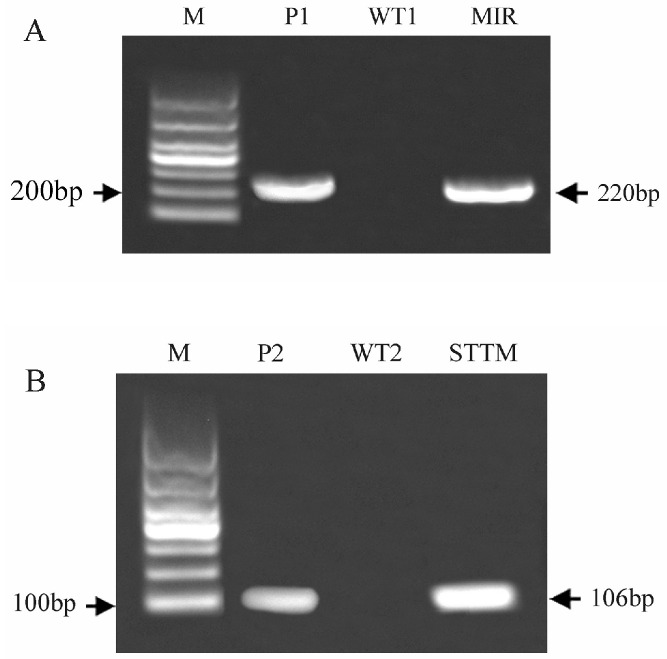
PCR identification of transgenic *Arabidopsis thaliana*. (**A**,**B**) show the PCR identification of the bvg-MIR156a and bvg-STTM-miR156a transgenic lines, respectively. M, 1000 bp DNA Marker; WT1 and WT2, wild-type Arabidopsis; MIR, overexpressed Arabidopsis; STTM, miR156a-silenced Arabidopsis; P1 and P2, bvg-MIR156a fragment and bvg-STTM-miR156a fragment, respectively.

To analyze the function of bvg-miR156a in plants, we initially cloned MIR156a, as well as STTM-miR156a, and constructed the overexpression vector pCAMBIA1300-35S-bvg-MIR156a (Figure 7A) and the microRNA-silencing vector pCAMBIA1300-35S-bvg-STTM-miR156a (Figure 7B). These constructs were then infiltrated into *Arabidopsis thaliana* using the flora dip method. Seeds were harvested to the T_2_ generation, producing two transgenic lines named MIR and STTM. DNA was extracted and subjected to PCR identification, with wild-type plants (WTs) serving as the negative control and bvg-MIR156a and bvg-STTM-miR156a as the positive controls. The results presented in Figure 8 confirm the successful construction of the transgenic lines.

### 2.8. Characterization of Transgenic Arabidopsis

**Figure 9:**
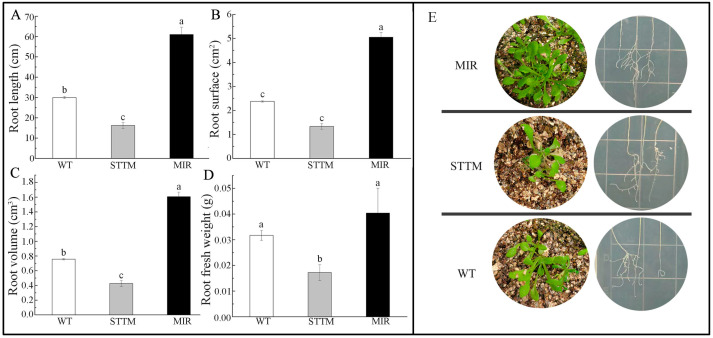
Phenotypic characterization of transgenic Arabidopsis: (**A**) root length; (**B**) root surface area; (**C**) root volume; (**D**) root fresh weight; (**E**) phenotypic performance of transgenic Arabidopsis. WT, wild-type Arabidopsis; STTM, microRNA-silenced Arabidopsis; MIR, overexpressed Arabidopsis; Different lowercase letters indicate the significance of differences between the detected plants, *p* < 0.05.

Subsequently, we evaluated the increased occurrence of transgenic Arabidopsis either overexpressing or silencing bvg-miR156a under both MS medium and soil conditions (Figure 9). The results showed that compared to STTM plants, MIR plants exhibited larger leaves and more lateral roots. Wild-type plants exhibited weaker root and leaf growth compared to overexpression plants but better than silenced expression plants. Specifically, the root length, root surface area, and root volume in MIR plants were 2.01-fold, 2.12-fold, and 2.12-fold higher than in WT, respectively, and 3.7-fold, 3.76-fold, and 3.78-fold higher than those in STTM plants, respectively. Additionally, the fresh weight of MIR roots increased by 27.4% compared to WT, while STTM roots decreased by 45.6% compared to WT. These findings indicate that silencing miR156a inhibits lateral root growth, whereas overexpressing miR156a promotes it.

## 3. Discussion

### 3.1. Effect of Nitrogen Deficiency on Growth and Subcellular Structure of Sugar Beet Roots

Nitrogen is considered the most crucial nutrient limiting sugar beet crop production, with nitrogen deficiency often resulting in reduced yields [24]. Plant roots exhibit various adaptive strategies to regulate nutrient uptake and respond to stressful environments [25]. In this experiment, we observed phenotypic changes in sugar beet seedlings subjected to nitrogen starvation ranging from 12 h to 7 days and found that low-nitrogen stress increased the total root length and branching to improve nitrogen acquisition, thereby allowing the plants to take up more nitrogen. During short-term nitrogen deficiency, there was a noticeable increase in dry matter accumulation in the roots, consistent with observations by Davidson et al., who noted that organisms generally tend to allocate biomass to roots under stress conditions [26]. Sattelmacher et al. believe that the growth of crop roots is influenced by the balance of carbohydrates and nitrogen [27]. According to the principle of proximal distribution, low nitrogen limits aboveground growth, while carbon availability limits underground growth. Consequently, in nitrogen-poor environments, aboveground bioaccumulation is restricted, directing carbon resources to be predominantly allocated to the roots’ growth and development, thus facilitating enhanced nitrogen absorption [28].

Generally, root cells with an adequate nitrogen supply exhibit well-defined cell walls and membranes, along with intact nuclei, mitochondria, and other structures, indicating active and healthy cellular conditions [29]. In this experiment, we observed that as the duration of LN stress increased, the cell walls gradually contracted, membrane structures vanished, and the number of mitochondria and other organelles decreased, indicating progressive cell deterioration. Mitochondria are crucial organelles that are involved in plants’ carbon and nitrogen metabolism. Under low-nitrogen stress, it has been indicated that a large amount of reactive oxygen species (ROS) are generated, leading to mitochondrial damage, tricarboxylic acid cycle inhibition, and reductions in photorespiration and nitrogen assimilation rates, which negatively affects plants’ nutrient utilization [30].

### 3.2. microRNA Expression Diversity Involved in Low-Nitrogen Stress of Beet Roots

MicroRNAs are short non-coding RNAs that interact with target mRNA transcripts in plants, modulating the gene expression at both the post-transcriptional and translational levels and participating in various stress responses [31]. Yang et al. demonstrated that miR827 regulates drought tolerance in potatoes by controlling the stomatal density [32]. Ma et al. identified miR827 as a key regulator of phosphate homeostasis [33]. Downregulation of miR167 significantly increases the grain weight and filling rate and is involved in auxin signal transduction and adventitious root formation [34]. Overexpression of miR319 enhances salt and drought tolerance [35]. MicroRNA expression levels vary among different species, and even within the same species, different stress conditions can induce distinct changes in microRNA expression [36].

In this study, it was found that LN-responsive root microRNAs were primarily involved in starch and sucrose metabolism, tyrosine metabolism, pyrimidine metabolism, amino sugar and nucleotide sugar metabolism, and isoquinoline alkaloid biosynthesis. Starch serves as the main form of carbohydrate storage in organisms and is a crucial carbon source for plants, predominantly stored in roots [37]. Sucrose, on the other hand, acts as an important signaling molecule in starch synthesis [38], significantly contributing to sugar accumulation in beets. Tyrosine plays a vital role in plant nutrition, with its derivatives engaging in the respiratory chain and functioning as electron transporters in various life processes [39]. In addition, tyrosine is also a precursor for phenylalanine synthesis, which is vital for plants’ secondary metabolism [21]. The carbon and nitrogen metabolisms constitutes the fundamental metabolic pathway in plants. During nitrogen starvation, microRNAs regulate specific target genes to maintain the balance of carbon and nitrogen metabolism, thereby alleviating the impacts of stress and aiding in plant recovery [40].

### 3.3. miR156 Improved the Potential Adaptability of Beet to Low-Nitrogen Stress

Exploring the relationship between microRNAs and their target genes provides insights into how they defend against external stress. Among the 22 DEMs, the microRNA regulatory network revealed that miR156a, with its conserved mature sequence, targets SPL (squamosa promoter-binding protein-like) transcription factors (104889216 and 104897537). In *Arabidopsis thaliana*, miR156 targets and regulates 11 SPLs, with SPL9 and SPL10 being highly expressed in roots, while SPL3 was predominantly expressed in flowers, fruit pods, and leaves, influencing flowering time regulation. Yu et al. demonstrated that exogenous auxin application in Arabidopsis promotes miR156 expression and enhances lateral root development [41]. Levy et al. highlighted miR172’s role in regulating SPL transcription factors, indicating a potential synergistic effect between miR156 and miR172 on lateral root growth [42].

Thus, we investigated the effects of overexpressing and silencing bvg-miR156a in transgenic Arabidopsis. The results indicated that the overexpression of bvg-miR156a promoted root length, lateral root formation, and increased fresh root weight, whereas silencing bvg-miR156a inhibited lateral root development and reduced biomass. This suggests that bvg-miR156a is involved in root growth regulation, thus influencing the entire plant growth cycle beyond just root development, which is similar to observations in potatoes [43]. In *Arabidopsis thaliana*, plants with high levels of miR156 exhibit increased branching and accelerated leaf development, attributed to sequential alterations in miR156 and its squamosa promoter-binding protein-like (SBP/SPL) targets. In these processes, miR156 and its targets (SPLs) are responsive to auxin signaling to function in plants’ organ development [44]. Therefore, in sugar beet, miR156 may directly or indirectly positively influence roots’ morphological development by regulating the SPL expression, thereby enhancing the plant’s adaptability to nitrogen deficiency.

## 4. Materials and Methods

### 4.1. Plant Materials and Cultivation

The low-nitrogen-tolerant sugar beet germplasm ‘780016B/12 superior’ [21] used in this experiment was obtained from the National Beet Medium-term Gene Bank in China (Harbin). Seeds with intact kernels were first sterilized with 75% alcohol and then soaked overnight in 2‰ thiram solution. After thorough rinsing with distilled water, the seeds were evenly sown in vermiculite and allowed to grow for approximately one week. Once seedlings developed two cotyledons, they were transferred to a Hoagland hydroponic system with a nitrogen concentration of 5 mmol/L for cultivation. The light intensity was 200 μmol/(m^2^·s), with a 25 °C/16 h day and 18 °C/8 h night cycle [21]. Low-nitrogen (0.5 mmol/L N) stress was introduced after the emergence of the second pair of true leaves for 12 h, 24 h, 3 d, 5 d, and 7 d. The control group maintained a nitrogen concentration of 5 mmol/L.

### 4.2. Determination of Root Morphological Indices and N Content

Three seedlings with consistent growth were selected for each replicate. The roots were rinsed with deionized water, and their fresh weight (FW) was measured using an analytical balance (sensitivity 0.001 g). The root morphology of the sugar beets was scanned and recorded using an Epson automatic scanner 1680 (Seiko Epson Corp, Tokyo, Japan). WinRhizo analysis software (WinRhizo Regent Instrument Canada Inc., Quebec, QC, Canada) was then used to analyze the root length, root surface area, root volume, effective bifurcation number, and other traits. The root surfaces were dried with filter paper, then placed at 105 °C for 15 min, followed by drying at 70 °C until a constant weight was reached. Then, the dry weight (DW) of the roots was measured. The dried samples were ground and analyzed [21] for nitrogen content using Kjeldahl^TM^ 8100 (Distillation Unit, FOSS, Copenhagen, Denmark) nitrogen analyzer.

### 4.3. Ultrastructural Observation of Sugar Beet Roots

Root tips (1–2 mm in length) from both the control and low-nitrogen groups were separately collected and immediately fixed in a glutaraldehyde solution, followed by postfixation with osmium tetroxide. After dehydration in ethanol and acetone, the samples were embedded in resin, sectioned using an ultramicrotome, and examined using transmission electron microscopy (Hitachi High-Tech, Tokyo, Japan, HitachiH-7650).

### 4.4. cDNA Library Construction, RNA Sequencing, and Data Analysis

Transcriptome sequencing was performed on sugar beet roots exposed to low-nitrogen stress (LN_R, 0.5 mmol/L) for 12 h, with 5 mmol/L N (CV_R) used as the control. RNA was extracted using RNA-easy Isolation Reagent (Vazyme R701, Vazyme, Nanjing, China). RNA integrity and purity were assessed using agarose gel electrophoresis and quantified using a Qubit Flex (Thermo Fisher Scientific, Shanghai, China), followed by evaluation with an Agilent 2100 (Agilent Technologies, Beijing, China). Libraries were prepared using the small RNA sample, which involved adding adapters to the 3′ and 5′ ends of microRNAs, followed by reverse transcription for cDNA synthesis. Target DNA fragments were separated via PAGE gel electrophoresis post-PCR amplification and retrieved to form cDNA libraries. Illumina-HiSeq^TM^ 2500 (Illumina, San Diego, CA, USA) sequencing was then performed on these libraries based on their effective concentrations and desired data yield [45]. Data processing included initial screening of raw data to remove low-quality reads, reads with undetermined base information, contamination with 5′ adapters, and reads lacking 3′ adapters and inserts, as well as trimming 3′ adaptors and polyA tails to produce clean reads. Sequences longer than 18 nt were used for subsequent analysis [46], as plant microRNAs typically range from 18 to 30 nt. MicroRNAs within this size range were aligned using Bowtie to analyze their distribution on the reference sequence [47]. Reads matching the reference sequence were compared against specified sequences in miRbase (https://www.mirbase.org/, accessed on 10 June 2022) to obtain detailed information on the sequence, length, and frequency of microRNAs in each sample. rRNA, tRNA, snRNA, and snoRNA sequences from Rfam [48] were employed to annotate the sequenced microRNAs and obtain clean microRNA sequences.

### 4.5. Differentially Expressed MicroRNA (DEM) Analysis and Transcriptome Data Validation

Statistical analysis of the known microRNA expression in each sample was conducted, with expression normalization achieved using TPM [49]. Differential expression analysis of the biological replicates was performed using DEseq2 [50]. DEMs with a q value <0.05 were selected for further analysis (Table S2). GO [51] and KEGG [52] analyses were carried out to elucidate the functional pathways enriched by DEMs under low-nitrogen stress. Subsequently, six DEMs were randomly selected for qRT-PCR verification. As shown in Table S3, U6 was chosen as the internal reference, and primers for qRT-PCR were designed using Primer Premier software (v5.0, www.premierbiosoft.com/primerdesign/, accessed on 2 July 2022), employing the stem-loop method [53] for cDNA reverse transcription primer design (Vazyme, miRNA 1st Strand cDNA Synthesis Kit (by stem-loop), MR101). The syntheses of the first-strand cDNA and qRT-PCR were conducted according to the kit instructions (Vazyme, miRNA Universal SYBR qPCR Master Mix, MQ101).

### 4.6. Prediction of DEM Target Genes and Construction of PPI Network

The target genes of mature microRNAs (Table S4) in sugar beet were predicted using Targetfinder (R1.6) [54], with an expected value threshold set to 3, and annotated via NCBI. The PPI network was constructed using the STRING v11.0b online database (https://version-11-0b.string-db.org/, accessed on 21 September 2022), and Cytoscape v3.9.1 (http://www.cytoscape.org/, accessed on 15 October 2022) was employed for data visualization.

### 4.7. Bioinformatics Analysis of bvg-miR156a

Mature miR156a sequences from *Zea mays* L., *Oryza sativa* L., *Glycine max* L., *Solanum tuberosum* L., *Nicotiana tabacum* L., *Brassica napus* L., and *Saccharum officinarum* L., used for microRNA analysis, were retrieved from the miRbase (https://www.mirbase.org/, accessed on 11 January 2023). The sugar beet miR156a sequence was aligned with sequences from these species. Phylogenetic tree analysis was performed using Mega11 (v11.0.13, https://www.megasoftware.net/dload_win_beta, accessed on 15 January 2023) with the ClustalW algorithm and the neighbor-joining method [55]. Bootstrap values were set to 1000, and the results were visualized using ITOL (https://itol.embl.de/, accessed on 15 January 2023) [56]. The mature sequence of bvg-miR156a was analyzed for conserved sequences using WebLogo3 (https://weblogo.threeplusone.com/, accessed on 20 January 2023) [57]. WebLogo parameters were set as follows: logo size medium, unit bits, and Y-axis spacing 2.0, with all other parameters set to default.

### 4.8. bvg-MIR156a and bvg-STTM-miR156a Plant Expression Vector Construction

The bvg-MIR156a sequence was synthesized by Nanjing GENE CREATE. Specific primers (Table S3) containing *BamH* I and *Xba* I cleavage sites were used for amplification and then ligated into the linearized pCAMBIA1300-35S plant expression vector (MIR) via homologous recombination. To construct the silencing expression vector, the U in the bvg-miR156a mature sequence was replaced with T, and a 48 nt linker sequence was used to connect two copies of the two miR156a mature sequences. *BamH* I and *Xba* I cleavage sites were added at both ends, with an additional CTA triplet inserted between the 10th and 11th bases. The designed silencing vector sequence was synthesized by Nanjing GENE CREATE and ligated into the linearized pCAMBIA1300-35S vector (STTM). Subsequently, these vectors were transformed into *Agrobacterium tumefaciens* GV3101 for genetic transformation.

### 4.9. Acquisition, Identification, and Phenotypic Trait Analysis of Transgenic Arabidopsis thaliana

The *Agrobacterium* culture was centrifuged at OD_600_ = 1.0 to collect the bacterial cells, which was then suspended in a sucrose osmotic solution containing a surfactant. Genetic transformation was performed on 5–6-week-old bolting *Arabidopsis* using the floral dip method. Infection was carried out weekly and repeated twice, followed by growth under conditions of 22 °C, 100 µmol/(m^2^·s) light intensity, a 16 h/d photoperiod, and 70% humidity in a growth chamber until seed harvest. T_2_ seeds were used for screening on an MS medium with chloramphenicol. DNA was extracted from the wild-type (WT) and T_2_ transgenic Arabidopsis (MIR: overexpressing and STTM: silenced) using the CTAB method and then amplified with specific primers in PCR (Table S3) using wild-type Arabidopsis DNA as a negative control and bvg-miR156 and STTM-miR156 fragments as positive controls. Root performance analysis was conducted as described in Section 4.2.

### 4.10. Statistics and Analysis

Each experiment was replicated three times. The data were processed using Excel and analyzed using SPSS statistical software (version 26.0, SPSS, Chicago, IL, USA). Significance was assessed by independent sample *t*-tests and one-way analysis of variance (ANOVA). All significance analyses were conducted at *p* < 0. 05. Origin 2021 and Adobe Photoshop 2019 were used for visualization.

## 5. Conclusions

This study explores the morphological, ultrastructural, and molecular changes in sugar beet roots under nitrogen stress. Although nitrogen deficiency limits beet growth and causes cellular damage, sugar beet roots actively respond to such nitrogen starvation by increasing their biomass, root length, and surface area to regulate nutrient uptake and assimilation product distribution. During these processes, 11 differentially expressed microRNAs, primarily participating in sugar synthesis and secondary metabolic pathways, likely play roles in the roots’ acclimation to a low nitrogen supply. Notably, miR156a, which is involved in root morphogenesis, appears to fulfill a key regulatory role in nitrogen use efficiency with its target gene *SPL*. In the future, exploring the functional roles of these microRNAs and their interactions with other regulatory networks might lead to novel strategies for improving crop performance under nutrient-limited conditions.

## Data Availability

SRA records will be accessible at the following link after the indicated release date: https://www.ncbi.nlm.nih.gov/sra/PRJNA1071583, will be accessed on 20 June 2026.

## Supplementary Materials

The following supporting information can be downloaded at https://www.mdpi.com/article/10.3390/ijms25169027/s1.
